# Impact of white matter lesions on clinical progression of Alzheimer's disease when accounting for regional neurodegeneration

**DOI:** 10.1002/alz70856_102768

**Published:** 2025-12-26

**Authors:** Nishita Paruchuri, John R Jacoby, David H Salat

**Affiliations:** ^1^ Massachusetts General Hospital Dept. Radiology, Boston, MA, USA; ^2^ Athinoula A. Martinos Center for Biomedical Imaging, Massachusetts General Hospital, Harvard Medical School, Charlestown, MA, USA; ^3^ Neuroimaging Research for Veterans Center, VA Boston Healthcare System, Jamaica Plain, MA, USA; ^4^ Massachusetts General Hospital, Boston, MA, USA; ^5^ Harvard Medical School, Boston, MA, USA; ^6^ Athinoula A. Martinos Center for Biomedical Imaging, Department of Radiology, Massachusetts General Hospital, Harvard Medical School, Charlestown, MA, USA

## Abstract

**Background:**

Alzheimer's disease (AD) is defined by a distinct neuropathological signature, yet symptom severity can vary among individuals with similar levels of pathology. For example, some individuals with the same degree of AD pathology exhibit severe dementia, while others retain relatively higher cognitive function, a phenomenon referred to as cognitive resilience. However, the mechanisms underlying this resilience require further investigation. Microvascular pathology, measured as white matter hyperintensities (WMH), typically considered independent of primary AD pathology, has been shown to influence disease progression. However, previous studies have not examined the impact of WMH after accounting for regional variation in neurodegeneration.

**Method:**

We leveraged data from the Alzheimer's Disease Neuroimaging Initiative (ADNI) to explore the role of WMH in cognitive status and resilience and the progression from mild cognitive impairment (MCI) to AD. Individuals with a baseline diagnosis of MCI were selected and stratified by sex (*n* = 413‐442 depending on the analysis). Within sex, participants were stratified into ‘high’ and ‘low’ cognitive performance groups using the ADNI cognitive composite scores based on residuals from cognition and individual brain region volume relationships after adjusting for age. Temporal regions were analyzed in relation to memory performance (ADNI‐Mem), while frontal regions were analyzed in relation to executive functioning (ADNI‐EF2). Analyses were conducted separately for men and women.

**Result:**

There was a significant difference in WMH volume in ‘high’ compared to ‘low’ performance groups in men but not in women. Effects remained after accounting for age and the volumes of temporal and frontal brain regions. In both men and women, individuals in the ‘low’ performance groups had significantly greater probability of AD conversion across the longitudinal interval.

**Conclusion:**

These results suggest a sex difference, with WMH burden serving as a stronger predictor of baseline cognitive status and AD conversion in men than in women. The findings also indicate that microvascular disease, quantified as WMH, contributes to cognitive variation with lower WMH being associated with greater resilience in MCI‐to‐AD progression beyond structural integrity. These results highlight the crucial role of vascular health in preserving functional independence in individuals with AD pathology.

